# The Effect of Spinal Cord Stimulation Frequency on the Neural Response and Perceived Sensation in Patients With Chronic Pain

**DOI:** 10.3389/fnins.2021.625835

**Published:** 2021-01-21

**Authors:** Gerrit Eduard Gmel, Rosana Santos Escapa, John L. Parker, Dave Mugan, Adnan Al-Kaisy, Stefano Palmisani

**Affiliations:** ^1^Saluda Medical Pty Ltd., Artarmon, NSW, Australia; ^2^Guy’s & St. Thomas’ NHS Foundation Trust, London, United Kingdom

**Keywords:** spinal cord stimulation, neural stimulation, stimulation sensation, stimulation frequency, evoked compound action potential (ECAP)

## Abstract

**Background:**

The effect of spinal cord stimulation (SCS) amplitude on the activation of dorsal column fibres has been widely studied through the recording of Evoked Compound Action Potentials (ECAPs), the sum of all action potentials elicited by an electrical stimulus applied to the fibres. ECAP amplitude grows linearly with stimulus current after a threshold, and a larger ECAP results in a stronger stimulus sensation for patients. This study investigates the effect of stimulus frequency on both the ECAP amplitude as well as the perceived stimulus sensation in patients undergoing SCS therapy for chronic back and/or leg pain.

**Methods:**

Patients suffering with chronic neuropathic lower-back and/or lower-limb pain undergoing an epidural SCS trial were recruited. Patients were implanted according to standard practice, having two 8-contact leads (8 mm inter-electrode spacing) which overlapped 2–4 contacts around the T9/T10 interspace. Both lead together thus spanning about three vertebral levels. Neurophysiological recordings were taken during the patient’s trial phase at two routine follow-ups using a custom external stimulator capable of recording ECAPs in real-time from all non-stimulating contacts. Stimulation was performed at various vertebral levels, varying the frequency (ranging from 2 to 455 Hz) while all other stimulating variables were kept constant. During the experiments subjects were asked to rate the stimulation-induced sensation (paraesthesia) on a scale from 0 to 10.

**Results:**

Frequency response curves showed an inverse relationship between stimulation sensation strength and ECAP amplitude, with higher frequencies generating smaller ECAPs but stronger stimulation-induced paraesthesia (at constant stimulation amplitude). Both relationships followed logarithmic trends against stimulus frequency meaning that the effects on ECAP amplitude and sensation are larger for smaller frequencies.

**Conclusion:**

This work supports the hypothesis that SCS-induced paraesthesia is conveyed through both frequency coding and population coding, fitting known psychophysics of tactile sensory information processing. The inverse relationship between ECAP amplitude and sensation for increasing frequencies at fixed stimulus amplitude questions common assumptions of monotonic relationships between ECAP amplitude and sensation strength.

## Introduction

It is commonly accepted that electrical stimulation of afferent cutaneous sensory fibres in the dorsal columns provides pain relief in neuropathic pain conditions. This concept, developed from the seminal paper by Melzack and Wall (1965) has since been refined and expanded upon. Although the exact mechanisms of action of spinal cord stimulation (SCS) are still debated, the efficacy in treating chronic neuropathic pain has been demonstrated in a plethora of studies and SCS has become a widely-used therapy across the globe (Kapural et al., 2015; North et al., 2016; Deer et al., 2018; Thomson et al., 2018; Mekhail et al., 2019; Russo et al., 2020).

In its original form, SCS used stimulus pulses of a few 100 μs repeated at a frequency of 50 Hz to activate the dorsal columns, eliciting a tingling sensation (paraesthesia), and inducing pain relief. Over the past decade, alternative stimulus waveforms have been proposed, ranging from square pulses delivered at higher frequencies (1–10 kHz), or a series of short bursts of pulses delivered at a given frequency, both delivering pain relief without necessarily eliciting a paraesthesia sensation (De Ridder et al., 2010; Kapural et al., 2015). There is however scant neurophysiological investigation into the mechanism of any of these waveforms to date.

**FIGURE 1:**
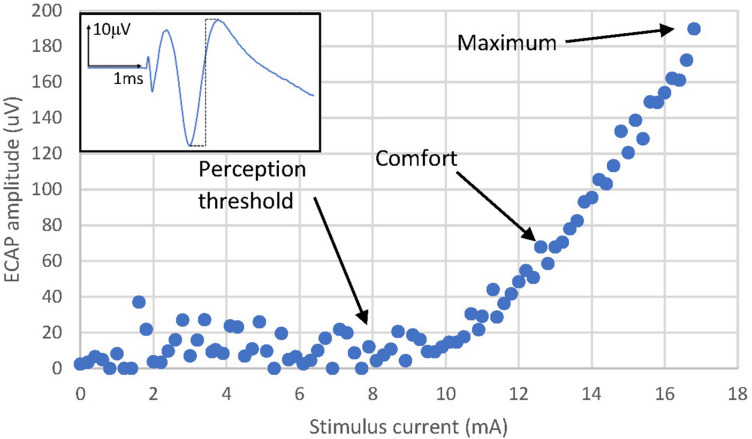
Activation plot example from Patient 13. The amplitude of the ECAP is calculated as the peak-to-peak amplitude of the first negative lobe and second positive lobe of the ECAP (dashed black line of the example ECAP shown in the figure inset). The ECAP amplitude increases linearly with stimulus current after reaching an activation threshold. Likewise, the sensation perceived also increases in strength from a threshold which normally is close to the activation threshold. Note that perception thresholds and ECAP thresholds can differ to a relatively small degree. Noise and the ability to record single fibre action potentials reliably is one factor, a potential psychophysical element cannot be ruled out. Sensation threshold was reached at 7.8 mA, a comfortable stimulation level was reached at 12.8 mA and the stimulus sensation was deemed maximal (starting to be uncomfortably strong) at 17 mA. Note that the threshold and slope of the activation plot for a particular patient with a fixed stimulating configuration varies with posture and other physiological changes (Parker et al., 2012, 2013; Mekhail et al., 2019; Russo et al., 2020).

Recent technological advances have now confirmed dorsal column activation during SCS treatment by recording Evoked Compound Action Potentials (ECAPs) in real time from the human spinal cord. The recorded ECAPs are a direct measure of the number of fibres activated by each SCS pulse (Parker et al., 2012; Mekhail et al., 2019; Russo et al., 2020). This lifts a major barrier to exploring the electrophysiological effect of SCS on its target. Initial publications have shown how the ECAP elicited by SCS increases linearly with stimulus current after a certain threshold is overcome (example in Figure 1) (Parker et al., 2012, 2013; Mekhail et al., 2019; Russo et al., 2020). Further, at frequencies in the 10–200 Hz range, the SCS-induced paraesthesia increases linearly with the amplitude of the ECAP, and this ECAP propagates at velocity consistent with that of sensory Aβ fibres typically encoding non-nociceptive sensation (Mekhail et al., 2019; Russo et al., 2020). This relationship between stimulus and ECAP amplitude is relatively well understood and models exist that describe how an extracellular stimulus pulse activates axons (Rattay, 1999; Parker et al., 2018).

The effect of varying the stimulus pulse width on fibre activation has also been widely explored and characterised for over a century (Weiss, 1901). Larger pulse width leads to lower thresholds for fiber activation (resulting in larger ECAPs for constant stimulus current) and stronger stimulus-induced paraesthesia (these reports also show that changing the pulse width can modulate the fibre recruitment order in some cases) (Yearwood et al., 2010; Holsheimer et al., 2011; Molnar and Barolat, 2014).

The effect of the stimulus frequency on fibre activation and stimulus-induced paraesthesia during continuous stimulation is less well understood and studied. Some insight can be gained from the study of the effect of pulse trains on nerve excitability. This has been investigated since the first half of the 20th century, mostly in relation to conduction block from repeated stimulation of demyelinated fibres. In 1935, Gasser reported long-lasting hyperpolarising after-potentials in normal fibres after repeated stimulation, a finding later confirmed by others (Gasser, 1935; Jansen and Nicholls, 1973; Bostock and Grafe, 1985; Morita et al., 1993). More recently, continuous stimulation over 10 min at 8, 20, and 30 Hz has been shown to cause a prolonged depression in fibre excitability in the human median nerve (Kiernan et al., 2004). The depression in excitability was stronger for 30 and 20 Hz stimulation than 8 Hz stimulation. This data remains quite limited with only three frequencies tested and their inability to perform any threshold assessment during stimulation. These researchers all concluded that electrogenic sodium/potassium pumps were the main contributors to the long-lasting hyperpolarisation after repeated stimulation.

We present here a study designed to investigate the role of stimulus frequency on dorsal column activation during ongoing SCS in subjects with chronic pain, exploring the effects of frequency on both the ECAPs amplitude and the subject’s perception of the stimulation.

## Materials and Methods

### Experiment Setup

**FIGURE 2:**
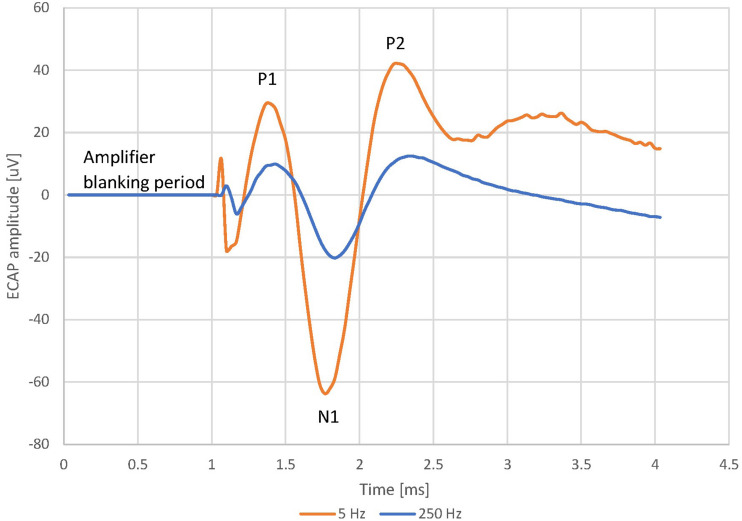
Evoked Compound Action Potential in Patient 13 at 5 and 250 Hz, 7.9 mA stimulus current and 100 us pulse width. The frequency sweep was started at 5 Hz and increased up to 250 Hz before the stimulus became uncomfortable. The patient reported a sensation of 2/10 at 5 Hz and 9/10 at 250 Hz. The voltage between 0 ms (time reference that marks the start of the stimulating pulse) and 1 ms is 0 uV. This “blanking period” is due to the neural amplifiers of the recording system being blanked to avoid saturation while the stimulation pulse is delivered.

Twenty patients undergoing epidural SCS trial for chronic neuropathic pain in the lower back and/or lower limbs were recruited to participate in the study; written informed consent was sought for all recruited patients, and the study protocol received Ethical Committee approval (REC Reference: 18/LO/0344, April 2018). All patients underwent an SCS trial with two 8-contact leads (8 mm inter-electrode spacing) inserted in the posterior epidural space according to standard practice with an overlap of 2–4 contacts around the T9/T10 vertebral interspace. Both lead together thus spanned about three vertebral levels. During two routine follow-up visits, a custom external stimulator capable of simultaneous real-time recording from each contact was connected to the lead (Saluda Medical MCS Mk II, similar to that described by Parker et al. (2013). Neural recordings (ECAPs) were obtained from all electrodes not used for stimulation. The ECAP amplitude was always calculated as the peak-to-peak amplitude (voltage) between the first negative lobe (N1) and the following positive lobe (P2) of the ECAP (Figure 2).

Stimulation and recording were first tested at different locations along the leads at 30 Hz, using biphasic, tripolar pulses with pulse widths of either 30, 100, or 240 μs (see Table 1). The stimulation location and pulse width that gave the best signal-to-noise ratio and was comfortable for the patient was then chosen to investigate the effect of varying the stimulus frequency. In these so-called “frequency sweeps”, the stimulus frequency was varied while maintaining all other stimulus parameters (such as stimulus current and pulse width) constant. We aimed to perform the widest possible frequency sweeps and the experiments contain stimulus frequencies ranging from 2 to 455 Hz. At the low end, the system limits us to a minimum frequency of 2 Hz. At the high end, patient comfort and the ability to obtain a non-truncated ECAP limits the maximum stimulus frequency.

**TABLE 1:** List of patients with available data.

**Patient number**	**Stimulation vertebral level**	**Pulse width (μs)**	**Sweep direction (range) [Hz]**	**Current (mA)**	**Sensation intensity available**
2	T9/T10	30	Increasing (2–100)	14.8	No
3	T11	30	Decreasing (16–300)	12.5	Yes
4	T11	30	Increasing (4–112) and decreasing (20–500)	37 (increasing) and 35 (decreasing)	Yes
5	T11	100	Increasing (5–141)	11.4	Yes
7	T10/T11 and T9	240	Increasing (10–130) and increasing (6–170)	3.2 (T10/T11) and 4.2 (T9)	No (increasing) and yes (decreasing)
10	T10/T11	100	Increasing (10–84)	18.8	Yes
11	T11/T12	100	Increasing (10–147)	6	Yes
12	T11	30	Increasing (10–60)	29.9	Yes
13	T11	100	Increasing (5–250)	7.9	Yes
14	T8	240	Increasing (5–65) and decreasing (29–152)	7.6 (increasing) and 7.5 (decreasing)	Yes
15	T11	240	Increasing (30–137)	6	No
16	T11	30	Increasing (10–455)	31.4	No
17	T11	240	Decreasing (16–294)	11.6	Yes
18	T10	240	Increasing (2–35) and decreasing (8–303)	8 (increasing) and 6.4 (decreasing)	Yes
19	T11	100	Increasing (20–78) and decreasing (3–120)	6.6 (increasing) and 7.3 (decreasing)	Yes
20	T10	240	Increasing (3–34) and decreasing (31–80)	5.3 (increasing) and 5.6 (decreasing)	Yes
					

As the stimulus frequency was varied, the perceived intensity of stimulation was reported by the patient using a scale from 0 to 10 (stimulation locations were typical for SCS and ranged from the lower back to the feet). Perceived stimulation intensity reporting was omitted in some patients. As patients proved to have different sensitivities to frequency changes, we tried to set the starting frequencies such as to maximise the ECAP amplitude while maintaining the stimulation at a comfortable level. Unless the signal-to-noise ratio was poor, or the patient was uncomfortable during testing, frequency sweeps were performed once by increasing the frequency from a low starting frequency and once by decreasing the frequency from a high starting frequency. The frequencies were changed by incrementing/decrementing manually, thus allowing us to respond to the patients’ reported sensation or maintaining stimulation constant for a few seconds or minutes to allow the patient to communicate effectively what they perceived. Both in the interest of time and to avoid habituation of the patient to various stimulus levels, we aimed to increment/decrement the frequencies about every second.

### Data Analysis

Averaging of ECAPs obtained from each set of stimulus parameters was used to improve the signal-to-noise ratio prior to measuring the amplitude (higher frequencies therefore leading to a larger number of ECAPs to average for the same time spent stimulating). In order to compare and aggregate the results across patients and experiments we normalised we normalised both the ECAP amplitudes and the reported sensation to their respective values at 30 Hz for each experiment. The ECAP values are reported as a percentage of their value at 30 Hz, and the sensation intensity scores are reported as absolute differences from the sensation level at 30 Hz.

To investigate the nature of the relationship between the ECAP amplitude and the stimulus frequency, the normalised ECAP amplitudes across all available experiments were aggregated and the average taken for each stimulus frequency. Various models (linear, polynomial and logarithmic) were fitted to the average normalised ECAP amplitude points to determine an approximate simple relationship. The same approach was taken for the sensation data.

## Results

Table 1 shows the stimulus parameters for each of the frequency sweeps reported in the study. Overall, 22 frequency sweeps from 16 patients were analysable. Stimulation sensation was reported in 18 frequency sweeps by 13 patients.

Data from this study revealed both a decrease in ECAP amplitude and an increase in stimulation-induced sensation intensity for increasing stimulus frequency. As illustrative example, Figure 2 shows the averaged ECAP at 2 distinct stimulus frequencies from patient 13. The ECAP amplitude is 124 μV at 5 Hz but only 32.6 μV at 250 Hz. Despite the smaller ECAP amplitude, the patient reported a sensation strength of 2/10 at 5 Hz and a strength of 9/10 at 204 Hz. The frequency sweep was stopped at 250 Hz as the stimulation was becoming too uncomfortable for the patient.

**FIGURE 3:**
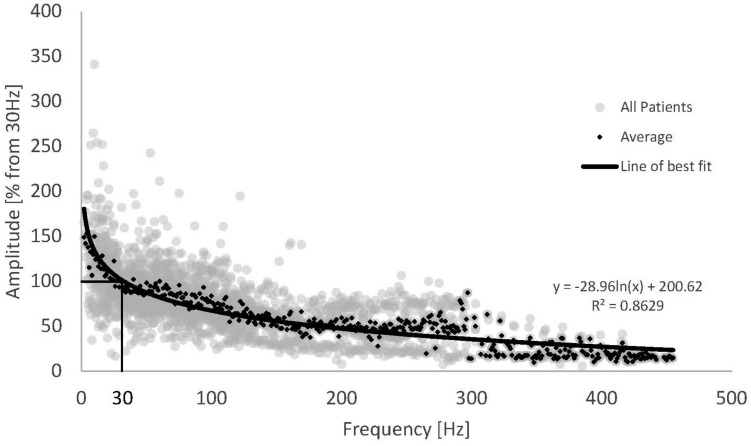
Normalised ECAP amplitude against stimulus frequency for all available data (grey dots) and the resulting average (black diamonds). Data from 16 patients and 22 frequency sweeps. A logarithmic fit was added to the average ECAP amplitudes.

The trend shown for Patient 13 above was observed across the whole patient cohort. Figure 3 shows the normalised frequency sweep data (all ECAP amplitudes normalised to their value at 30 Hz) for all patients and the average ECAP amplitude for each frequency across the whole dataset. There is a significant reduction in ECAP amplitude with the increase in the frequency of stimulation, and the amplitude drop is most pronounced at lower frequencies, indicating a non-linear relationship with frequency. A logarithmic line of best fit through the average ECAP amplitude gives an R (Kapural et al., 2015) of 0.863.

**FIGURE 4:**
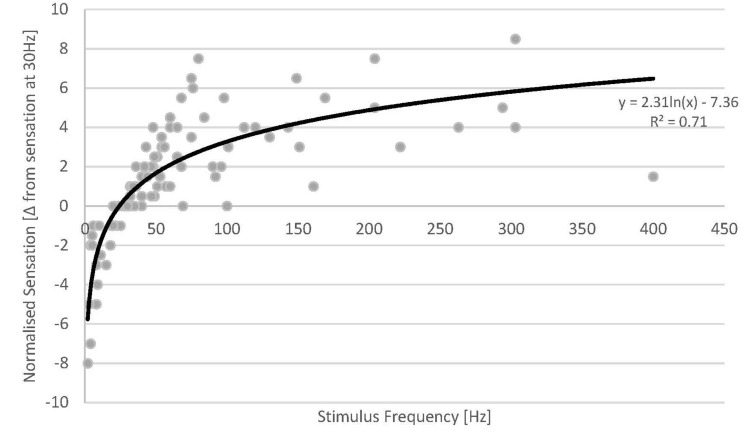
Normalised sensation of all patients versus stimulus frequency (grey dots) (a total of 18 sweeps across 13 patients). Fewer data points are available at high stimulus frequencies as the stimulus sensation often became too strong for the patients at the given stimulus amplitude used. A logarithmic fit was added (solid line).

Across the patient cohort with available sensation data, all patients reported an increase in stimulation sensation with increasing stimulus frequency (Figure 4). Despite the inherent inaccuracy of scoring a continuous variable such as stimulation sensation intensity in a discrete fashion, our results display a consistent behaviour across the whole cohort of studied subjects. The increase in sensation was more prominent for lower frequencies (2–50 Hz), a behaviour which seems to follow a logarithmic trend of opposite sign to that observed in the ECAP amplitude. As the stimulation sensation became stronger at higher frequencies, the patient tolerance limited the maximum frequency for each experiment (see Table 1), therefore, data at high frequencies becomes sparse.

The data from our experiments demonstrate that the stimulation sensation is dependent on stimulus frequency. Given these results, we investigated whether the ECAP amplitude could account for some of the unexplained variance from the logarithmic fit against stimulus frequency. We created a new variable by multiplying the ECAP amplitude by the frequency (units of V/s) and plotted the difference in sensation against this variable. We found virtually no difference in the R (Kapural et al., 2015) between these two fits (0.707 vs. 0.705). This results from the interesting property that, as both the ECAP amplitude and the stimulus sensation seem to have logarithmic relationships with respect to frequency, they would themselves be linked by a linear relationship (as long as the stimulus amplitude remains constant).

## Discussion

This study presents the first report in humans of the effect of stimulus frequency (between 2 and 455 Hz) on both the recruitment of dorsal column fibres and the patient’s perception of the stimulus sensation during SCS. When the SCS current intensity is kept fixed, the ECAP amplitude and reported sensation strength responded in opposite ways to changes in frequency, with the ECAP amplitude decreasing while the sensation strength increased with increasing frequency.

Our findings stand in stark contrast with the well-known property that ECAP amplitude and stimulus perception both increase with increasing stimulus amplitude past a threshold (all other parameters kept constant) (Parker et al., 2012; Russo et al., 2020). It also stands in opposition with the known effects of pulse-width variations on fibre recruitment where larger pulse widths decrease the activation threshold for the fibres (Yearwood et al., 2010; Holsheimer et al., 2011; Molnar and Barolat, 2014). So, while both pulse width and pulse amplitude have an overall effect of activating more fibres per pulse, leading to a stronger stimulus sensation, increasing the stimulus frequency decreases the number of fibres activated by each pulse but still increases the perceived stimulus sensation.

### ECAP Amplitude and Frequency

It is commonly thought that changes in nerve excitability from repeated activation is mediated by electrogenic sodium/potassium pumps (Gasser, 1935; Jansen and Nicholls, 1973; Bostock and Grafe, 1985; Morita et al., 1993; Kiernan et al., 2004). The role of sodium/potassium pumps in fibre threshold change has been established experimentally using agents acting principally on these pumps (such as ouabain which inhibits the Na/K-ATPase), a clear mechanism of action has however not been established. An alternative hypothesis postulates that potassium accumulation in the peri-axonal space is the main driver of excitability changes with repeated stimulation. Experimental evidence shows that axons are able to follow pulse trains only up to a certain frequency (between 50 and 130 Hz in deep brain stimulation literature) (Jensen and Durand, 2009; Zheng et al., 2011). Perhaps more relevant for SCS are similar findings of frequency-dependent axonal conduction block in rats’ dorsal column fibres at kilohertz frequencies (Crosby et al., 2017). Crosby et al. demonstrated that at higher frequencies, fibres were unable to follow every stimulating pulse, resulting in asynchronous firing with activation of the fibres only achieved for a subset of stimulus pulses. Various models have associated this frequency-dependent intermittent conduction block with the accumulation of potassium ions in the peri-axonal space caused by repeated firing (Bellinger et al., 2008; Guo et al., 2018). Although most of these studies are centred around deep brain stimulation targets, the principles of axonal firing and potassium accumulation directly apply to SCS.

As the ECAP is the sum of action potentials generated in various fibres by a stimulus pulse, our findings of reduced ECAPs size when the frequency of stimulation is increased could be explained by either an increase in fibre threshold or by the intermittent block of the same fibres. Whether this is mediated by electrogenic pumps or ion accumulation cannot be determined from the recordings.

### Stimulation Sensation and Frequency

The sensation strength data collected from the patients clearly demonstrate that the stimulation sensation is dependent not only on the ECAP amplitude but also on the stimulus frequency, highlighting the complex relationship between paraesthesia sensation and fibre recruitment patterns. Our findings are unique in the human SCS literature. Previously, Abejón et al. (2016) demonstrated that, maintaining a fixed pulse width in subjects already implanted with SCS, an increase in frequency reduces the current needed (constant current system) to reach the sensory threshold. This is in accordance with our results, showing an increase in sensory perception parallel to the increase in stimulation frequency while the current intensity is kept unchanged. However, the group speculated that the increase in SCS frequency leads to the recruitment of a greater number of neurons, while we have clearly demonstrated that neural recruitment (i.e., ECAPs amplitude) per pulse decreases. This highlights the importance of real-time ECAPs recording in humans in expanding our knowledge of the effects of SCS on neural tissue and replacing speculation with quantitative data.

The typical activation plot shown in Figure 1 is demonstrative of *population coding* of the perceived stimulus sensation strength, i.e., the more fibres are recruited by each pulse, the stronger the perceived stimulus. However, our data highlight that *frequency coding* also plays a large role in cutaneous sensory coding, where the firing rate of the sensory neurons encodes the perceived stimulation amplitude. The existence of both neural coding schemes for sensory fibres is well established (Kandel et al., 2013), but the way in which an artificial firing pattern such as that produced by SCS affects both the neural recruitment and the sensory experience is less well understood. Most striking is the apparent weight of the frequency coding component and the population coding component. Despite a decrease in the ECAP amplitude with increasing stimulus frequency, patients report an increase in the perceived stimulus strength; in our experiments, frequency coding seems to outweigh population coding in the explored parameter range.

The exact coding scheme used to convey tactile sensory information is not known. Whilst both population coding and frequency coding do play a role, frequency-based neural codes have been found to predict tactile stimulus strength better than population-based neural coding schemes (Muniak et al., 2007; Bensmaia, 2008). A first attempt at combining both neural codes has been made by Graczyk et al. (2016) which define an activation charge rate, taking into account both the total charge of electrical pulses and their frequency, to encode tactile stimulus sensation strength.

It is important to note that SCS differs greatly from activation of mechanoreceptors by mechanical stimuli on the skin. Whereas mechanoreceptors react in different ways to constant or varying mechanical stimuli, generating different firing patterns, SCS activates axons directly. It is quite possible that the perceived stimulation intensity from SCS cannot be well understood from psychophysical and neurophysiological studies of mechanical stimuli. A major shortcoming of this study has been the inability to separate the effect of ECAP amplitude from stimulus frequency on the patient’s perceived stimulus sensation. Further work should explore the effects of stimulus frequency and stimulus amplitude on both recruitment and sensation simultaneously (by, for example, conducting a current sweep at multiple stimulus frequencies). A qualitative assessment of the stimulus perception should be also undertaken, as various stimulus patterns will be interpreted in different ways by the higher cortical centres.

As discussed above, the frequencies explored here were limited by patient sensation, with higher stimulation frequencies giving stronger stimulation sensation at constant stimulus amplitude. Primary sensory afferents are refractory for about 1 ms after stimulation, thus our stimulation parameters did not involve refractory properties of the fibres. This study did not aim to investigate therapeutic efficacy of various stimulus frequencies, but it is noted that tonic SCS typically only stimulates at frequencies between about 10 and 100 Hz. Interestingly, stimulation frequencies at 1 kHz and above and “burst” stimulation paradigms (sets of five pulses in close succession) have been shown to provide therapeutic stimulation at amplitudes below perception thresholds (Kapural et al., 2015; Deer et al., 2018; Thomson et al., 2018; Karri et al., 2020). We are currently designing experiments to investigate the effect of burst and supra-kilohertz stimulation on neural activation and the link to patient perception (or lack thereof).

## Conclusion

To our knowledge, this is the first report providing a quantitative assessment of the effect of stimulus frequency (<500 Hz) on the recruitment of dorsal column fibres and the resulting change in stimulation perception in the human spinal cord. It highlights the importance of quantitative measures to help understand the mechanisms of action of SCS and neuromodulation in general as proxy measures for neural activation (such as stimulus sensation) are prone to misinterpretation.

## Data Availability Statement

The datasets presented in this article are not readily available because the data is the property of Saluda Medical. The data can be made available upon request at the discretion of Saluda Medical. Requests to access the datasets should be directed to GG, gerrit.gmel@saludamedical.com.

## Ethics Statement

The studies involving human participants were reviewed and approved by the NHS Health Research Authority. The patients/participants provided their written informed consent to participate in this study.

## Author Contributions

GG, RSE, JP, DM, AA-K, and SP contributed to the design of the study. GG, RSE, and SP executed the study and analysed the data. GG wrote the first draft of the manuscript. All authors read, revised, and approved the submitted version.

## Conflict of Interest

GG, RSE, DM, and JP were employed by the Saluda Medical Pty Ltd. The remaining authors declare that the research was conducted in the absence of any commercial or financial relationships that could be construed as a potential conflict of interest.
